# Formulating Geopolymer Mortars through Construction and Demolition Waste (CDW) Recycling: A Comprehensive Case Study

**DOI:** 10.3390/ma16237304

**Published:** 2023-11-24

**Authors:** Stefania Manzi, Luca Baldazzi, Andrea Saccani

**Affiliations:** Department of Civil, Chemical, Environmental and Materials Engineering, University of Bologna, Via Terracini 28, 40131 Bologna, Italy; luca.baldazzi4@unibo.it (L.B.); andrea.saccani@unibo.it (A.S.)

**Keywords:** geopolymers, construction and demolition waste, mechanical properties, porosity

## Abstract

The overall amount of construction and demolition waste (CDW) is steadily increasing due to urbanization-related phenomena in metropolitan cities. Only a small fraction is recycled to produce new concrete, a practice that would avoid the exploitation of natural aggregates. Furthermore, the Portland cement production process causes the release of high values of CO_2_ emissions into the atmosphere, increasing the global warming potential. For these reasons, materials alternative to ordinary Portland cement (OPC) are becoming more and more widespread, such as alkali-activated materials, which, when used with recycled aggregates, could become environmentally friendly substitutes for traditional concrete. During this study, various mix designs were formulated for alkali-activated metakaolin mortars containing recycled concrete aggregates. Their properties in the fresh and hardened states were analyzed. The main problem encountered was the presence of the adhered mortar layer on the recycled concrete aggregate. This layer not only caused a detrimental effect deriving from the increase in open porosity but also activated competitive reactions which partially compromised the alkali activation of metakaolin. All these phenomena deteriorated the final compressive strength of the composites containing recycled aggregates, which after 28 days, was around 20 MPa for samples with 12.5% of replacement of natural aggregate and 15 MPa for those with 25% of replacement, corresponding to a reduction of 35% and 50% compared to the standard sample without replacement, respectively.

## 1. Introduction

### 1.1. Environmental Impact of CDW

In recent years, the diffusion of concrete in construction has constantly increased. This phenomenon is promoted both by the growth of the global population and by the urbanization of the population who originally lived in the countryside towards new metropolitan cities in search of better job opportunities. Rapidly developing countries such as China, India, Brazil and South Africa are the most affected by these events. Consequently, concrete production has reached approximately 30 billion tons of annual global consumption, which corresponds to 8 tons/year of cement consumed by each human being [1,2,3]. An increase in world cement production of approximately 50% is also estimated from 2017 to 2050 [4]. It must be added that the production of the binder used in concrete (ordinary Portland Cement—OPC) is a cause of high CO_2_ emissions and involves the consumption of natural resources such as marly rocks. In fact, it is estimated that to produce one ton of OPC, it is necessary to extract 1.5 tons of raw rocks and emit 0.8–1 ton of CO_2_ [3], to which must be added the emission of NO_x_ and SO_2_, which causes an impact of 5–8% [5] on anthropogenic emissions responsible for the greenhouse effect [6]. Furthermore, this production is also quite inefficient since 40% of the energy is wasted during the production process due to the high processing temperature (1400 °C) [6]. Finally, concrete has a negative impact on the environment even at the end of a building’s service life. After the building’s demolition, concrete is mostly sent to landfill, sometimes turning into a special waste due to dangerous materials such as asbestos that can be mixed during the demolition. It is estimated that in 2012, 830 Mt [6] of recycled concrete waste (CDW) was produced in the EU (equal to 25–30% of the total waste produced in the EU) [7], which subsequently decreased to 374 Mt in 2018 [8]; 600 Mt was produced in the USA in 2018, and 2.5 billion tons in China in 2015 [8], of which 40% was concrete waste [9], which suffered a strong increase in the following years. In China, only 30% of CDW waste is recycled and CDW is generally used as landfills in suburban areas, creating soil and air pollution [10]. The global production of CDW is estimated at 10 billion tons/year [8]. Concrete makes up 68% of the total amount of CDW [11]. Another sector involved in the reuse of recycled materials is that of road construction since the most commonly used materials, asphalt and concrete, have a strong impact on the environment [12]. These data underline the danger associated with the disposal of CDW linked to the risk of contamination of the environment with toxic substances such as polychlorinated biphenyls, asbestos and heavy metals.

In the strategies for the design of sustainable buildings of the World Green Building Council (2019), there is a plan to achieve a 40% reduction in carbon emissions by 2030, and of 100% by 2050 [3]. To achieve these objectives, it is therefore necessary to immediately foresee strategies aimed at progressively reducing the production of greenhouse gases in every production process. Directive 2018/851/EU establishes that Member States take initiatives to promote the selective demolition of CDW, ensure the treatment of hazardous substances and increase the recycling of such materials. Therefore, the recycling type becomes very important to achieve the objectives of the circular economy and avoid low-value recycling that does not allow for new recycling processes [13]. By improving the efficiency of collection and transforming waste into a resource, the EU sets the objectives of reducing imports of raw materials and facilitating the green transition based on a circular economy model, ensuring sustainable and inclusive economic growth (Europe 2020 strategy) able to increase the synergies between the economy and environmental policies [14].

### 1.2. Geopolymers and CDW

Geopolymers or alkali-activated materials are inorganic polymers with a chain structure formed by aluminum and silicon ions, which can be synthesized via geopolymerization (mixing aluminosilicates with highly alkaline solutions of activators) [3,15]. Geopolymerization is composed of four parts: dissipation, dissolution and diffusion, polycondensation, and dehydration. In the first step, oxide minerals are dissipated in an alkaline environment (break of Al-O and Si-O bonds); in the second step, the tetrahedral monomers are released and later diffused; in the third step, there is the formation of a 3D amorphous matrix of alumino-silicates—Si-O-Al-O; finally, after the dehydration, the geopolymers achieve their mechanical strength. The advantages of geopolymers are a low environmental footprint (reduction in CO_2_ emissions by 9–80% compared to OPC), resistance to acids and high temperatures, rapid hardening, good mechanical properties, corrosion resistance and a long lifetime as a result of the formation of a compact structure [3,5,16,17,18,19]. Furthermore, the early strength of geopolymers makes them suitable for precast industries, creating a structure in a short amount of time and with less chance of breaking during transport [20]. For all these reasons, in recent years, more and more research has focused on the development of new recipes based on geopolymers (replacing OPC) and CDW (replacing natural aggregate or as precursors of alkali-activated materials) [3,4,5,21,22,23,24,25].

CDW can be used as a substitute for the aggregate both in the coarse fraction and in the fine fraction. The main component of coarse recycled aggregate is concrete, followed by bricks, asphalt, and tiles [20]. The quality of the final concrete will depend on several factors including the properties of original CDW aggregates (cement concrete, water/cement ratio, and age of concrete), the degree of replacement, the pretreatment process, and their grain size distribution [11]. When CDW is used to replace the aggregates, the major problem consists of the interfacial transition zone (ITZ) between the recycled aggregate and the new matrix which presents micro-cracks caused by the old mortar remaining attached [26]. Furthermore, the recycled aggregates are more porous than natural stones; therefore, they will also have greater water absorption and lower density (reduction of around 6–10%), absorbing a higher amount of water during the mixing process, creating a decrease in the workability of the geopolymer mortars [4,25]. Possible solutions are (1) presoaking the aggregate before the mixing process or (2) the use of superplasticizers. The bond strength between the residual OPC mortar and the geopolymers in the ITZ is inversely proportional to the water/solid ratio [27]. The concrete ITZ between binder and aggregate is the weakest part of the matrix and it is where cracks develop under the load applied. Furthermore, its high porosity allows the sulfates, chlorides and other acids to enter, causing damage and decay. The geopolymer ITZ is, in contrast, very dense, achieving a higher strength and durability; however, to obtain a strong bond connection between the aggregate and the geopolymer matrix, a high concentration of alkali and soluble silicate is necessary [28]. Kirthika [29] found that increasing the amount of recycled fine aggregate coming from CDW negatively influences both the durability and the strength owing to the existence of old adhered mortar in the ITZ, and he found the optimum replacement to be 30%. To remove the old adhered mortar at the ITZ interface, some processes were tested to improve the performance of geopolymer products. One of these is the heating of the recycled aggregate (around 300 °C) to dehydrate the old mortar; sometimes, this treatment is followed by a rubbing process performed with steel balls [11]. Other treatments to remove it include the following: the “smart crusher”, a particular jaw crusher able to separate the coarse aggregate from concrete without breaking it; and a chemical attack with HCl, H_2_SO_4_ (more efficient) or C_2_H_4_O_2_. Different treatments have the aim of improving the properties of the recycled aggregate, such as the accelerated carbonation in the CO_2_ chamber to covert C-S-H in CaCO_3_; the Na_2_SiO_3_ and Li_2_SiO_3_ solutions that react with Ca(OH)_2_ to form C-S-H; and the inclusion of nanoparticles with an ultrasonic processor [11]. For the geopolymer mortars or concrete with the replacement of the natural aggregate, various tests were performed by changing the curing temperature, the molarity of the solution, and the treatment with which the aggregates are treated to remove the old adhered mortar in order to obtain products with better performance. Pawluczuk [4] previously treated the aggregate by subjecting it to a patented thermal (>650 °C) and mechanical treatment, thus obtaining good mechanical strengths for an activator concentration > 6 M and curing at 80 °C (which was the more impactful parameter on mechanical strength). However, the presence of a coarse aggregate from treated CDW improved the mechanical properties even at low curing temperatures (40 °C). Also, Panizza [30] tested the replacement of the natural aggregate with CDW coming from bricks, concrete or a mix of them in geopolymers with metakaolin and slag as precursors, coming to the conclusion that aggregates obtained only from bricks give products more porous but with a greater increase in strength, and that higher curing temperatures increase the mechanical properties. Furthermore, a substitution of CDW aggregates greater than 40% of the dry weight leads to a decrease in mechanical resistance. Panizza [30,31] found a value of compressive strength of 38 MPa for geopolymer mortars obtained using metakaolin, blast furnace slag and fly ash as precursors, potassium silicate as an activator and CDW coming from bricks, tiles and concrete waste as aggregate. Volpintesta [32], using geopolymers with metakaolin and potassium silicate, completely replaced the natural aggregate with CDW (in the 0–8 mm fraction) coming from earthquake-demolished buildings in central Italy in 2016. This replacement produced a decrease in mechanical strength, an increase in open porosity and water absorption, and a delay in the hardening; nevertheless, even the samples with 60% replacement by weight of CDW had resistances comparable to those of common structural concretes (29 MPa). De Rossi [33] tested mortars obtained from fly ash and metakaolin as precursors, NaOH and Na_2_SiO_3_ as activators and CDW (mix of concrete and brick) as aggregate in a complete replacement of natural sand. The results obtained show good mechanical properties (40 MPa for compressive strength) but lower values of flowability. Nuaklong [34] tried to replace fly ash with metakaolin in concretes containing CDW as coarse aggregate coming from concrete samples collected in the laboratory and of known compressive strength (30–40 MPa) and fine natural aggregate coming from river sand. The results showed that this replacement with 30% metakaolin allowed for an improvement in the mechanical properties, water absorption, and porosity, and resistance to abrasion and acid attack. Akbarnezhad [35] tested the possibility of re-using the same geopolymer concrete as aggregate in replacement of the natural one to create a new recycled geopolymer concrete. The results gave values of compressive strength inferior by 12.9% for the natural aggregate compared to a compressive strength decrease of 28.1% for aggregates coming from recycled OPC. According to this study, geopolymer concretes can also be considered recyclable at their end of life. Even the performance of CDW geopolymers was recently tested by Giannopoulou [36], who discovered good thermal stability until 1050 °C. Finally, Akduman [37] tested geopolymer concrete to produce reinforced beams using CDW both as precursors (mix of bricks, roof tiles, glass, and concrete) and aggregate (maximum grain size 10 mm). The results show that the replacement of natural aggregate with recycled aggregate affects the strength due to ITZ formation; in contrast, the beams prepared using CDW-geopolymer binder but with natural aggregate have a similar behavior to that of the conventional concrete. Also, the mechanisms of failure are very similar.

As can be seen in this brief review, there are many ways to recycle CDW in association with geopolymer materials. However, it is important to remember that these products, in order to have an effective response on the market and therefore a generic beneficial impact on the environment (through the reduction in CO_2_ in the production process and lower consumption of natural resources such as river sands and marly rocks), must be easily made on site even by poorly qualified personnel and the overall cost of the finished product must be competitive. Some heat treatments on CDW risk being expensive, while curing at temperatures above 40 °C appears difficult to achieve on-site construction, limiting this product to prefabrication only. A problem associated with the correct mix design of CDW geopolymers is the difficulty in formulating an adequate recipe given that the parameters for the formulation are many: type of precursors, type and concentration of alkaline activators, SiO_2_/Al_2_O_3_ and Na_2_O/SiO_2_ molar ratios, water/solid ratio, type of CDW, pretreatment temperature of aggregate, mechanical pretreatment of aggregate, and curing time and temperature. To solve this problem Shen [38] tested three different machine learning models, creating a database of 164 mix proportions and 328 samples to predict the mechanical properties of CDW geopolymers.

The aim of this research project is precisely to investigate different formulations of alkali-activated mortars based on metakaolin containing different amounts of CDW as a partial replacement for the natural aggregate. The CDW was used without requiring any preventive thermal or mechanical treatments. Indeed, only a simple washing procedure with water was performed and tested for a series of samples and compared to the as-received aggregates. Moreover, basic curing at room temperature was performed. Such a treatment will not lead to the best mechanical properties but will have the lowest energy consumption, also allowing for on-site application. A preliminary test on the durability of the mortars was performed but a more complete characterization will be needed to evaluate the materials’ performance in demanding environments.

## 2. Materials and Methods

### 2.1. Materials

#### 2.1.1. Aggregates

**Figure 1:**
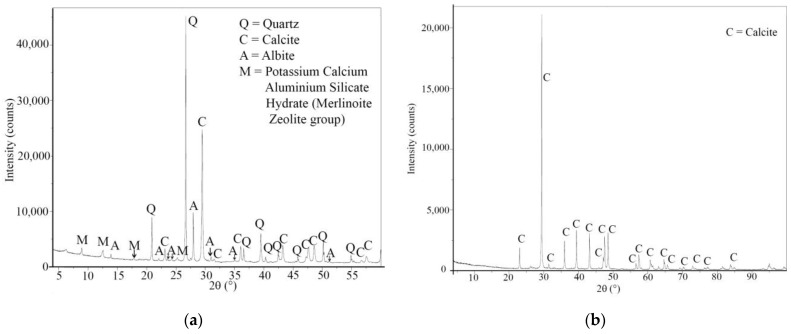
XRD pattern of (**a**) natural aggregate and (**b**) recycled aggregate.

The natural aggregate in the fraction 0–2 mm (F) was natural silica sand (SiO_2_ > 96 wt.%) with a fixed grain size distribution (d_max_ = 2 mm) according to EN 196-1 Standard [39]. The natural aggregate in the fraction 2–4 mm (N) was natural sand collected from the Frantoio Fondovalle quarry (Bologna, Italy). N aggregate was analyzed with the XRD technique (diffractometer Empyrean, Malvern Panalytical, detector PIXcel1D, Almelo, The Netherlands). A 300 g sample of both aggregates was crushed until reaching a specimen powder that passed through a 0.075 mm sieve, which was afterwards subjected to X-ray analysis. Figure 1a shows that the diffractogram of N is composed mainly of calcium carbonate, quartz and albite. In particular, quartz is present in the highest amount. The recycled aggregate (R) analyzed in this research comes from the demolition of the skeletal concrete structure of “Punta Perotti” real estate complex, which took place in 2006 in Bari (Italy) [40,41] and is formed only by concrete crumbles. Figure 1b shows that the diffractogram of R is composed only of calcium carbonate.

Water absorption of the N and R aggregate fractions of 2–4 mm was calculated according to UNI EN 1097-6 [42], and the values obtained are shown in Table 1.

**Figure 2:**
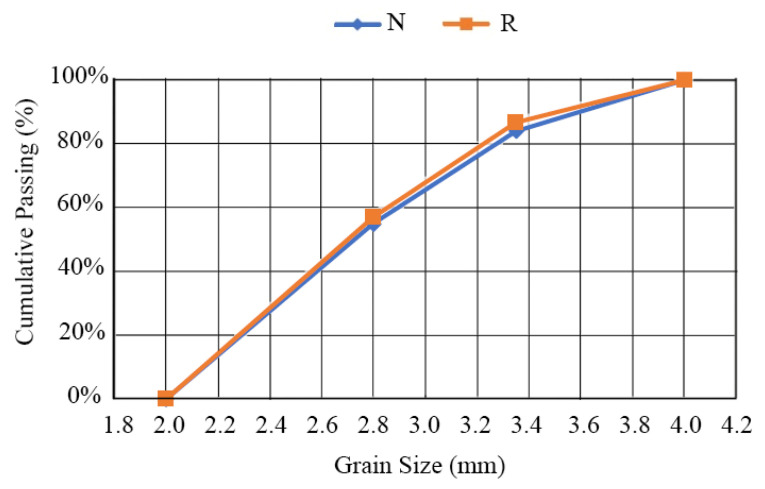
Grain size distribution of N and R aggregates.

N aggregates were also sieved to obtain the same grain size distribution of R (Figure 2).

**Figure 3:**
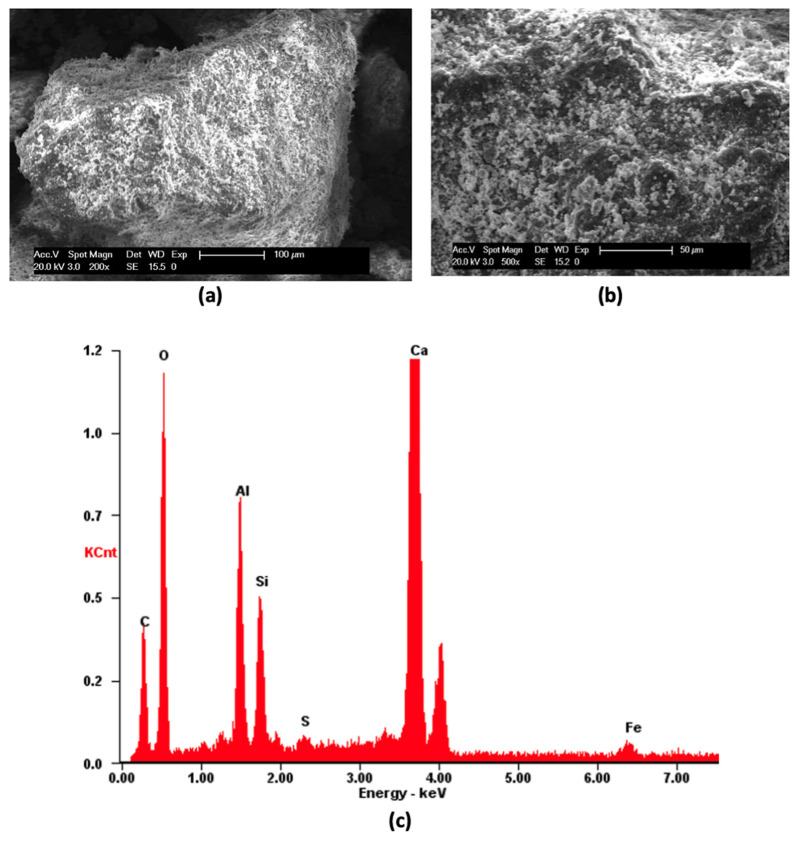
SEM images of R aggregate: (**a**) surface; (**b**) detail of surface; (**c**) EDAX analysis performed on R aggregate.

The R aggregate was also analyzed with a scanning electron microscope (SEM XL20 type, FEI Instruments, FEI, Hillsboro, OR, USA) after carbon sputtering (Figure 3a,b). The morphological aspect of the aggregates is not a smooth one, but powder-like fragments are clearly visible, covering the whole surface. The EDAX analysis (Figure 3c) shows the presence of energy peaks related to calcium, aluminum, silicon, iron and sulfur. The presence of calcium was expected from the results of the X-ray analysis, which revealed their calcite composition (Figure 1b), but the other elements derive from the Portland cement layer of the pristine matrix still adhering to the surface of the aggregate. These elements were not detected in the X-ray analysis on account of both their reduced quantity in the investigated samples and the amorphous character of the hydration gel.

#### 2.1.2. Binder

The precursor used is metakaolin (MTK, sourced from ARGECO Dévelopement, Toulouse, France).

#### 2.1.3. Activators

The activators are an 8M solution of NaOH (Merck, Darmstadt, Germany) and Na_2_SiO_3_ (with a water content of 56 wt.%, SiO_2_/Na_2_O ratio = 2.07, ρ = 1.53 g/cm^3^ produced by SS, Ingessil, Verona, Italy). The alkaline activators (NaOH + Na_2_SiO_3_) were previously mixed at a low speed and cooled until room temperature before the sample’s preparation.

### 2.2. Mix Design and Sample Preparation

Different mixes were studied (Table 2), starting from a reference mix design with 100% natural aggregates (A-Series) according to previous research [43], and the amount of recycled aggregates varied between 12.5 wt.% (B-Series) and 25 wt.% (C-Series) over the total content of aggregates.

Different aggregate conditions and mixing times were also tested (Table 3):

- The 1 series were prepared using unwashed aggregates in saturated-surface dry conditions. The aggregate and the amount of water necessary to obtain saturated-surface dry (SSD) conditions were mixed the day before.
- The 2 series were prepared using a washed recycled aggregate (R) in saturated-surface dry conditions (SSD). In particular, the R aggregate was previously washed in water, to remove the impurities present on the surface of the aggregate, a possible barrier to the adhesion between aggregate and matrix. The aggregate and the amount of water necessary to obtain saturated-surface dry (SSD) conditions were mixed the day before.
- The 3 series were prepared using aggregates in dry conditions. The aggregates (N and washed R) were added in dry conditions and the amount of water to obtain the saturated-surface dry conditions was added to the water of the recipe. An additional mixing time (i.e., 5 min) was also applied.

The geopolymer mortars with recycled aggregate were obtained by using a laboratory mortar mixer (5 L volume). Initially, metakaolin was placed in the vessel of the mixer and the activation solution (NaOH + Na_2_SiO_3_) with water was added. After 3 min of stirring, the natural sand (F and N) followed by the R aggregate were added in a continuous flux for 60 s. After 90 s of stoppage to remove the mortar from the wall of the vessel, a final mixing at high speed for 60 s was carried out. The total procedure lasted 5 min for samples noted with numbers 1 and 2. In contrast, for samples noted with number 3, the entire mixing process lasted 10 min to compensate for the addition of the aggregate in dry condition. From the fresh mortars, different samples were cast for each series: (a) 3 specimens of 40 × 40 × 160 mm to be subjected to physical and mechanical tests; and (b) 3 cylinders with 35 mm of diameter and 100 mm height to be subjected to water absorption via capillary test. After 24 h of storage at room temperature in sealed polyethylene bags, the mortar samples were demolded and later stored in the same conditions until tests.

All the recipes are reported in Table 2, while the aggregate characteristics and mixing times are reported in Table 3.

### 2.3. Methods

#### 2.3.1. Determination of Consistency

After the mixing process, the mortars were cast in a brass truncated conical mold (ϕ 70–100 mm, 60 mm height), according to EN 1015-3 [44]. Two perpendicular diameters of the collapsed mortar were measured, and the standard consistency (C) was thus calculated according to Equation (1).

C = 100·(d_m_ − d_0_)/d_0_, (1)

where d_m_ is the average diameter of the two perpendicular diameters and d_0_ is the lower diameter of the mold (i.e., 100 mm).

#### 2.3.2. Physical Properties

The mortars’ bulk density (ρ_b_) was determined by weighing the samples in dry, wet and hydrostatic conditions, according to the EN 772-13 Standard [45], at 28 days of curing. Total open porosity (OP) was calculated by determining the water absorption (WA) at atmospheric pressure according to EN 772-21 Standard [46] at 28 days of curing.

Three cylindrical samples (ϕ 35 mm, height 100 mm) were used for each series to measure the water absorption via capillary test, according to EN 15801 [47] after 28 days of curing. The amount of water absorbed was calculated using Formula (2), as follows:

Q_i_ = (m_i_ − m_0_)/A, (2)

where Q_i_ is the amount of absorbed water at time t_i_ (kg/m^2^), m_i_ is the weight of the sample at time t_i_ (kg), m_0_ is the weight of the dried sample (kg), and A is the absorption area in contact with water (m^2^).

#### 2.3.3. Mechanical Properties

Flexural (σ_f_) and compressive (σ_c_) strength were measured after 28 days of curing at room temperature and R.H. 60 ± 10% by means of a 100 kN Wolpert Amsler test machine (Wolpert, Neu Ulm, Germany) with a 5 mm/min displacement rate. Except for the reported parameters, the tests were performed according to the EN 196-1 Standard [39]. Dynamic elastic modulus (E_d_) was measured on 40 × 40 × 160 mm prisms after 28 days of curing before the compression test, with an ultrasonic pulse velocity system (Matest, Treviolo, Italy) according to EN 12504-4 [48] using Equation (3), as follows:

E_d_ = V^2^·ρ, (3)

where E_d_ is the dynamic elastic modulus (Pa), V is the pulse velocity (m/s), and ρ is the density of the sample (kg/m^3^).

#### 2.3.4. Microstructure

SEM analyses were performed by means of a SEM XL20 type (FEI Instruments, FEI, Hillsboro, OR, USA) scanning electron microscope equipped with an EDS X-ray detector. The fractured surfaces of the samples to observe were coated with graphite to ensure electrical conductivity. An accelerating voltage of 20 kV was applied during all measurements.

#### 2.3.5. X-ray Diffraction Analysis

XRD analysis was performed to evaluate the mineralogical composition of the geopolymer mortars. The diffraction patterns were obtained using an Empyrean Malvern Panalytical diffractometer (Almelo, The Netherlands) with a detector PIXcel1D equipped with Cu radiation operated at 40 kV and 20 mA. The pulverized samples were scanned between 0° and 90° 2θ.

## 3. Results and Discussion

Table 4 and Table 5 show the average diameter measured during the standard consistency test (d_m_), the final consistency (C) and the physical (ρ_b_, WA ad OP) and mechanical properties (σ_f_, σ_c_ and E_d_) of the samples. As can be observed, the consistencies (C) of the series noted as 1 and 2 are substantially equivalent to each other (70 ÷ 80%), and the highest value of consistency belongs to the C2 series (80%). On the contrary, series 3, where aggregates were previously washed but added in dry form, has the lowest value of C (45 ÷ 50%), even if, at the same time, no substantial difference can be observed among them. In particular, the values are comparable or even higher than those of the A1 references and this could be a good compromise in terms of workability. In all the tested conditions, there is no detrimental effect of the recycled aggregate on the investigated property. Concerning water absorption (WA) and open porosity (OP), on account of the higher porosity of the recycled aggregate detected via the previous determination of its density (ρ_rd_ = 1800 kg/m^3^), a direct proportionality between the increasing amount of natural aggregate replaced and the water absorption and the open porosity takes place. At the same time, a slight but progressive decrease in density is reported as the amount of R increases.

An increase in the recycled aggregate amount creates mortars with higher open porosity and lower values of mechanical strength. These results are consistent with those of another research [32]. For instance, it can be observed that in samples noted as 3, an increase in the R aggregate equal to 25% corresponds to an increase in OP of 15% and a decrease in compressive strength of about 55%. Mixes with unwashed aggregates (B1 and C1 samples) have a decrease in flexural strength, compared to the reference, of no more than 50%. Samples with washed aggregate have better performances, in particular at a low amount of substitution. The replaced mix at 12.5% has a decrease in compressive strength of approximately 28%; in contrast, the replaced mix at 25% has a decrease of 55%. As regards the compression strength values, it can be underlined that the different conditions (1, 2 or 3) did not show any remarkable differences, although the washed samples provide better results at the lowest amount (B2, B3) of R. Moreover, the saturated condition of the aggregate (B2) only slightly increases the mechanical properties of the samples if compared to the B3 condition. Indeed, the saturated condition should lead to the creation of a stronger ITZ, providing a remarkable effect as reported elsewhere [4]. This observation seems to underline that another feature must be contributing to the decrease in compressive strength. When the R amount increases (C samples), no positive effect of the washing process is found, and the results are almost the same in all the samples. The trend of the modulus is always decreasing as the R amount increases.

**Figure 4:**
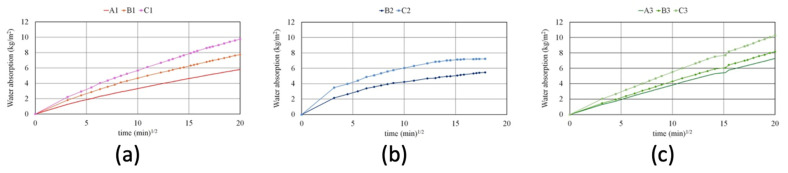
Water absorption via capillarity curves: (**a**) samples of the 1 series (A1, B1, C1) with unwashed aggregate in saturated-surface dry conditions; (**b**) samples of the 2 series (B2, C2) with washed aggregate in saturated-surface dry conditions; (**c**) samples of the 3 series (A3, B3, C3) with washed aggregate in dry conditions.

Analyzing the graphics of water absorption through capillarity (Figure 4), the samples containing recycled aggregates recorded high absorption rates. The increase in the rate of water absorption in the samples is proportional to the percentage of R aggregate substituted at all the investigated conditions (washed/unwashed, saturated or dry). This effect is mainly related to the higher porosity of the recycled aggregate and, as shown in Table 4, to the higher porosity of the composite. Condition 2 (washed sample and SSD) shows the lowest rates of absorption, suggesting that a beneficial effect of the washing process was found, by analyzing the mechanical properties, only in the B2 samples. The higher absorption rate hints at an increased permeability of the substituted mortars and this can lead to a decreased durability of the materials. Indeed, chlorides and sulphates could diffuse more rapidly into the structures. Specific tests should be carried out but a comparison with traditional substitutes mortars should also be performed.

**Figure 5:**
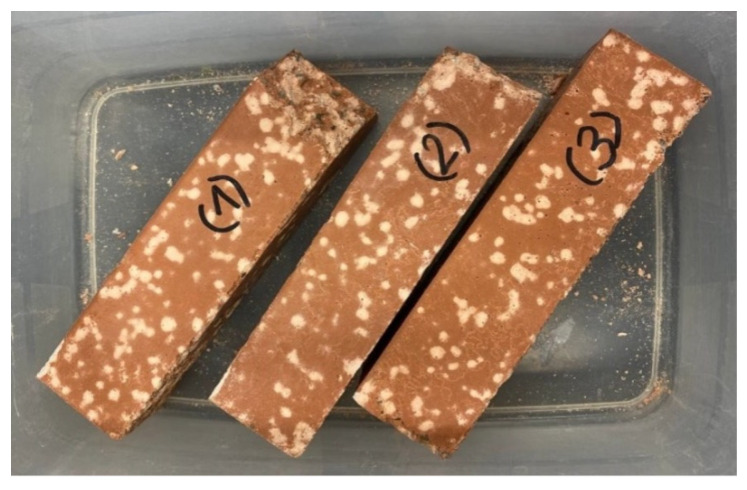
Image of three different mixes of C2 specimens (with R aggregates) with the presence of white stains a few days after the demolding procedure.

After some days of curing at room temperature, the 40 × 40 × 160 mm specimens with R aggregates showed the formation of white circular stains on the surfaces (Figure 5).

**Figure 6:**
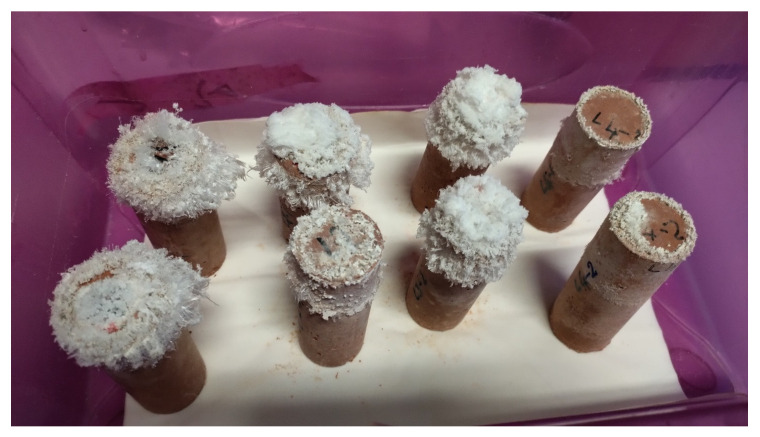
Efflorescence in the cylinders tested for determination of water absorption by capillary.

At the same time, in the cylindrical specimens submitted to the water absorption test, white efflorescence was found on the surface at the maximum level of capillary rise (Figure 6). Specimens A showed a quite limited extent of this phenomenon (samples on the right side of Figure 6).

**Figure 7:**
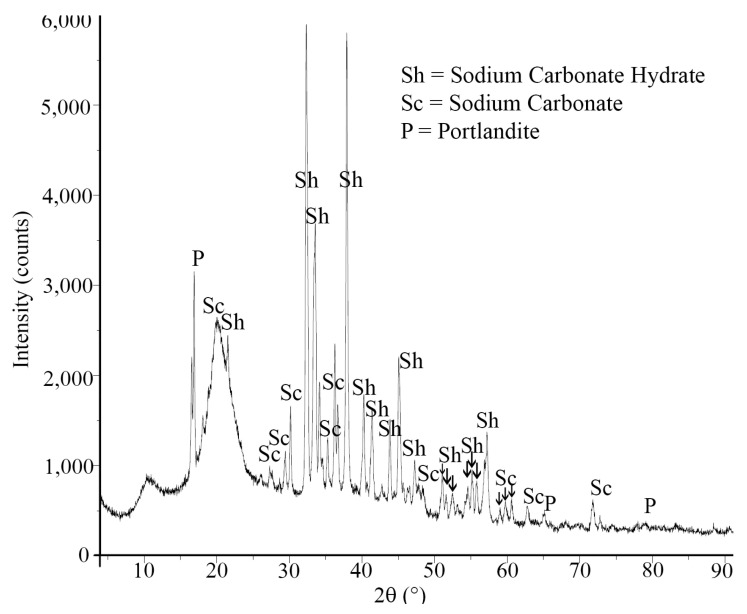
XRD performed on the filtered material of the recycled aggregate.

In order to analyze the role of the recycled aggregate in both events, samples of recycled aggregate were placed in an 8 M NaOH solution and stirred. After 24 h, the samples were filtered and, later, the fine retained material was dried to perform an XRD analysis (Figure 7). The results show the presence of sodium carbonate (Na_2_CO_3_) and sodium carbonate hydrate (Na_2_CO_3_·H_2_O) deriving from the alkaline solution, but also the presence of portlandite. The same results were found in the recovered liquid after drying. This proves that, in strong alkaline conditions, a part of the adhering cement mortar on the aggregate can be dissolved.

**Figure 8:**
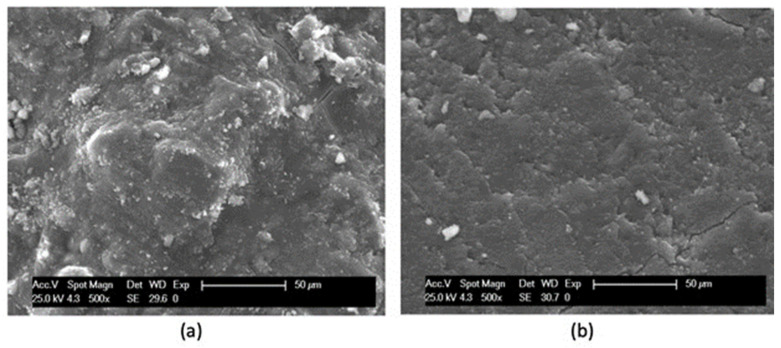
SEM analysis on geopolymer samples: (**a**) white stains near the aggregate surface of Figure 5; (**b**) geopolymer matrix (coming from the red zone of Figure 5).

**Figure 9:**
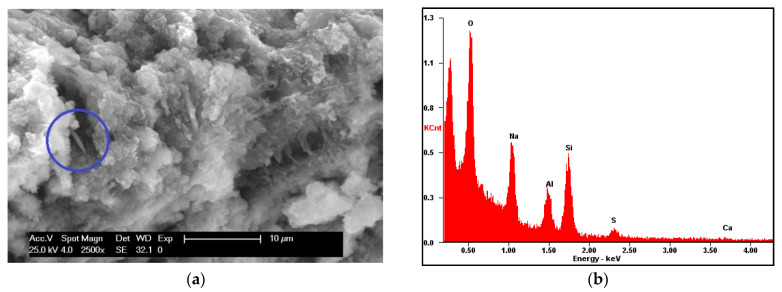
SEM analysis: (**a**) an hydration product (in the blue circle) of the reaction of cement in the matrix; (**b**) EDAX analysis close to the recycled aggregate’s surface.

Moreover, both the white stains and the red regions on the prismatic samples were observed separately with SEM analysis (Figure 8) on fractured composite surfaces of samples with an average dimension of 1.5 × 1.5 cm. A difference in the extent of the reactions in the matrix is envisaged. When the metakaolin activation occurs, a compact, almost shiny surface similar to glass is formed (Figure 8b). In the volume close to the recycled aggregate (Figure 8a), several separate metakaolin particles can still be distinguished, owing to an activation that did not completely occur. Near to the surface of the aggregate, it is also possible to observe the presence of hydration products deriving from the old cement mortar attached to the recycled concrete aggregate (SEM image of Figure 9a). It can be assumed that the still unreacted OPC particles present in the recycled aggregate entered into competition with the geopolymerization reaction, preventing the accomplishment of this one. A second feature detected (Figure 9b) is the presence of sulfur atoms in the area close to the recycled aggregates. The release of sulfur atoms coming from the old cement mortar can possibly be a second negative effect, again compromising the activation of the precursors.

**Figure 10:**
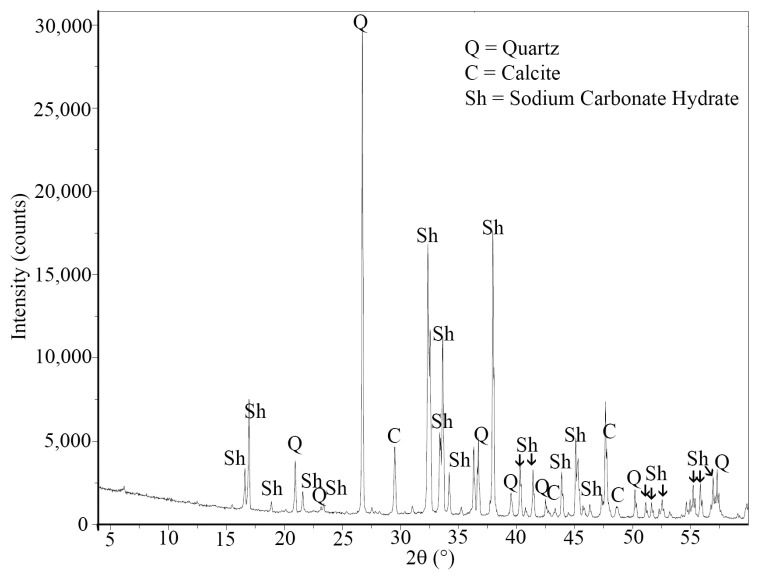
XRD patterns of the efflorescence detected on the cylinders with recycled aggregate.

Eventually, the white efflorescence found on the surface of the cylinders tested for water absorption via capillary at the maximum level of capillary rise (Figure 6) were analyzed. The XRD analysis (Figure 10) confirmed the presence of sodium carbonate hydrate (Na_2_CO_3_·H_2_O). Also, quartz (SiO_2_) and calcium carbonate (CaCO_3_) were detected. This first one may derive from the binder (metakaolin), while the second one may come from the recycled aggregate.

Recently, Tan [49] described the efflorescence in geopolymers as a “deep-seated disease” that can also create a deterioration in the mechanical properties. The process is described as a combination of leaching and carbonation. Internal free alkalis move to the surface due to pores, interacting with CO_2_ in the air, and, after carbonation, sodium carbonate (Na_2_CO_3_) is produced according to Formula (4), as follows:

2NaOH + CO_2_ + H_2_O → Na_2_CO_3_ + 2H_2_O. (4)

During the water absorption test, the presence of water in matrix derives from the capillary suction from the base. Later, this water can evaporate from the surface, forming whitish efflorescence products. This process can continue until the equilibrium condition between crystals and pore solutions is obtained. For Tan [49], the causes are the low Al availability of the precursors and the high values of water absorption. In our case, this can be considered further evidence of the compromised activation process near the recycled aggregates surfaces, causing a mismatch in the matrix composition. Unreacted sodium hydroxide, silica and calcium released from the aggregate can thus migrate and form the investigated efflorescence.

A deeper analysis of the reactions is, however, needed to completely understand the causes of the produced damage, in particular the role of the sulfur ions. It is important to underline that, in this research, the detrimental effect of the recycled aggregate on the mechanical properties is not only derived from the higher porosity of the aggregate or from the peculiar characteristic of the ITZ. Indeed, the same recycled aggregates tested on conventional Portland cement concrete [40,41] showed comparable or even higher concrete properties than the reference with natural aggregates.

## 4. Conclusions

The search for low-impact building materials promotes the substitution of Portland cement with alternative binders, as the one tested in this research. At the same time, the partial substitution of quarried aggregates with those derived from buildings demolition allows for the preservation of natural resources such as river sand and marble stones. Some problems may arise from the use of recycled aggregates since the overall porosity of the composites will increase and a weaker ITZ can be formed. The present research underlines that the problems involved in the use of recycled aggregates in geopolymer systems may not only arise from the porosity of the remaining cement’s old matrix, as usually found when exploiting the wastes in Portland cement composites. In this specific case study, the chemistry of the matrix surrounding the aggregate is altered, causing an unbalance in the ratio between metakaolin and activators. A possible cause, which deserves further detailed investigations, could derive from the diffusion of sulfur atoms from the adhering paste on the aggregate. A mild soaking process, performed on the aggregates before mixing, only slightly reduced this negative effect and a more complex treatment on the adhering mortar should thus be performed.

In the case of alkali-activated matrix, it would thus be important to carry out some preliminary analyses on the recycled concrete aggregates added (e.g., chemical and mineral composition, and water absorption). If these problems are solved, awareness regarding waste management and environmental protection will enable an increasingly widespread use of these materials in the building industry in the future.

## Figures and Tables

**Table 1:** Physical properties of natural and recycled aggregates.

Type	ρ_rd_ (kg/m^3^)	ρ_ssd_ (kg/m^3^)	WA (%)
N	2600	2700	2.2
R	1800	2000	12.7

N = natural aggregate 2–4 mm; R = recycled aggregate, ρ_rd_ = dry bulk density; ρ_ssd_ = saturated surface-dried density; WA = water absorption.

**Table 2:** Mix design of the sample mortars.

Series	% R	F (g)	N (g)	R (g)	MTK (g)	Na_2_SiO_3_ (g)	NaOH (g)	H_2_O (g)
A	0	675	675	0	450	225	45	65
B	12.5	675	506	169	450	225	45	65
C	25	675	338	338	450	225	45	65

F = natural aggregate 0–2 mm, N = natural aggregate 2–4 mm, R = recycled aggregate, MTK = metakaolin.

**Table 3:** Aggregate characteristics and mixing time.

Series	Recipe	N		R		Mixing Time
A1	A	SSD	unwashed	n.p.	n.p.	5 min
A3	A	dry	unwashed	n.p.	n.p.	10 min
B1	B	SSD	unwashed	SSD	unwashed	5 min
B2	B	SSD	unwashed	SSD	washed	5 min
B3	B	dry	unwashed	dry	washed	10 min
C1	C	SSD	unwashed	SSD	unwashed	5 min
C2	C	SSD	unwashed	SSD	washed	5 min
C3	C	dry	unwashed	dry	washed	10 min

N = natural aggregate 2–4 mm; R = recycled aggregate; SSD = saturated-surface dry conditions; n.p. = not present.

**Table 4:** Consistency and physical properties of geopolymer mortars.

Sample Name	% R	d_m_ (mm)	C (%)	ρ_b_ (kg/m^3^)	WA (%)	OP (%)
A1	0	170	70	2060 ± 0	9.0 ± 0.1	18.4 ± 0.1
A3	0	145	45	2040 ± 20	9.1 ± 0.1	18.7 ± 0.0
B1	12.5	177	77	2020 ± 0	9.8 ± 0.0	19.8 ± 0.0
B2	12.5	175	75	2040 ± 10	9.7 ± 0.2	19.9 ± 0.3
B3	12.5	150	50	2010 ± 10	9.9 ± 0.2	19.8 ± 0.2
C1	25	170	70	1990 ± 20	10.5 ± 0.2	21.0 ± 0.2
C2	25	180	80	2000 ± 10	10.7 ± 0.1	21.4 ± 0.2
C3	25	150	50	1950 ± 0	11.0 ± 0.1	21.5 ± 0.1

R = recycled aggregate; d_m_ = average diameter measured during the standard consistency test; C = consistency; ρ_b_ = bulk density; WA = water absorption, OP = open porosity.

**Table 5:** Mechanical properties of geopolymer mortars.

Sample Name	% R	σ_f_ (MPa)	σ_c_ (MPa)	E_d_ (GPa)
A1	0	6.3 ± 0.6	30.9 ± 1.9	26.1 ± 0.5
A3	0	5.8 ± 0.6	31.8 ± 0.7	26.8 ± 0.6
B1	12.5	3.5 ± 0.9	16.4 ± 2.5	21.1 ± 0.7
B2	12.5	4.7 ± 0.1	24.3 ± 1.9	20.5 ± 0.6
B3	12.5	4.8 ± 0.1	22.9 ± 1.7	19.6 ± 0.3
C1	25	3.3 ± 0.2	15.1 ± 1.6	15.8 ± 0.5
C2	25	3.7 ± 0.9	14.4 ± 1.8	18.1 ± 0.6
C3	25	3.3 ± 0.1	14.4 ± 0.7	14.9 ± 0.3

R = recycled aggregate; σ_f_ = flexural strength; σ_c_ = compressive strength, E_d_ = dynamic elastic modulus.

## Data Availability

The authors declare the availability of the data reported in this paper.
